# Statistical and bioinformatic analysis of hemimethylation patterns in non-small cell lung cancer

**DOI:** 10.1186/s12885-021-07990-7

**Published:** 2021-03-12

**Authors:** Shuying Sun, Austin Zane, Carolyn Fulton, Jasmine Philipoom

**Affiliations:** 1 Department of Mathematics, Texas State University, San Marcos, TX USA; 2 Department of Statistics, Texas A&M University, College Station, TX USA; 3 Department of Mathematics, Schreiner University, Kerrville, TX USA; 4 Department of Mathematics, Applied Mathematics, and Statistics, Case Western Reserve University, Cleveland, OH USA

**Keywords:** Methylation, Hemimethylation, Lung Cancer, Bioinformatics, Epigenetics

## Abstract

**Background:**

DNA methylation is an epigenetic event involving the addition of a methyl-group to a cytosine-guanine base pair (i.e., CpG site). It is associated with different cancers. Our research focuses on studying non-small cell lung cancer hemimethylation, which refers to methylation occurring on only one of the two DNA strands. Many studies often assume that methylation occurs on both DNA strands at a CpG site. However, recent publications show the existence of hemimethylation and its significant impact. Therefore, it is important to identify cancer hemimethylation patterns.

**Methods:**

In this paper, we use the Wilcoxon signed rank test to identify hemimethylated CpG sites based on publicly available non-small cell lung cancer methylation sequencing data. We then identify two types of hemimethylated CpG clusters, regular and polarity clusters, and genes with large numbers of hemimethylated sites. Highly hemimethylated genes are then studied for their biological interactions using available bioinformatics tools.

**Results:**

In this paper, we have conducted the first-ever investigation of hemimethylation in lung cancer. Our results show that hemimethylation does exist in lung cells either as singletons or clusters. Most clusters contain only two or three CpG sites. Polarity clusters are much shorter than regular clusters and appear less frequently. The majority of clusters found in tumor samples have no overlap with clusters found in normal samples, and vice versa. Several genes that are known to be associated with cancer are hemimethylated differently between the cancerous and normal samples. Furthermore, highly hemimethylated genes exhibit many different interactions with other genes that may be associated with cancer. Hemimethylation has diverse patterns and frequencies that are comparable between normal and tumorous cells. Therefore, hemimethylation may be related to both normal and tumor cell development.

**Conclusions:**

Our research has identified CpG clusters and genes that are hemimethylated in normal and lung tumor samples. Due to the potential impact of hemimethylation on gene expression and cell function, these clusters and genes may be important to advance our understanding of the development and progression of non-small cell lung cancer.

**Supplementary Information:**

The online version contains supplementary material available at 10.1186/s12885-021-07990-7.

## Background

Lung cancer is a leading cause of death in the United States; more patients die of lung cancer than of breast, prostate, and colon cancers combined. The American Cancer Society predicts that in 2021 alone there will be 235,760 new cases of lung cancer diagnosed and 131,880 deaths in the United States [1]. The five-year survival rate of lung cancer is much lower than many other prominent cancers such as breast, colorectal, and prostate, as only 19.4% of patients survive beyond 5 years of having the disease. The rate of survival can be as high as 57.4% when the cancer is still localized. However, the majority (57%) of patients are diagnosed in late stages, and the five-year survival rate of these patients is only 5.2% [2].

In order to diagnose and treat lung cancer, it is important to identify genetic and epigenetic biomarkers. One important epigenetic biomarker or event is DNA methylation. It is the covalent bonding of a methyl group (−CH_3_) to a CpG site in a mammalian cell; this is an epigenetic alteration to the DNA, meaning the DNA sequence does not change. A CpG site is the nucleotide base cytosine bonded to the base guanine by a phosphate (5′-CpG-3′) [3]. The correlation between methylation patterns on specific CpG sites and gene expression has been studied as methylation enhances or mutes particular genes [4]. DNA methylation patterns are maintained and changed mainly through two dynamic processes: methylation maintenance and de novo methylation [5, 6]. Methylation maintenance allows for preservation of methylation patterns across replication generations, maintaining valuable methylation levels. De novo methylation occurs on symmetrically unmethylated CpG sites and increases methylation levels over cell generations [5]. Demethylation is the action of a methyl group being removed from a CpG site, and it can be observed in two forms: passive and active [6]. Passive demethylation is an error during maintenance methylation, resulting in bare or hypomethylated CpG sites on the nascent strand, whilst the parent strand is methylated. In contrast, active demethylation is the deliberate removal of a methyl group from a CpG [7].

Both demethylation and de novo methylation can lead to the development of hemimethylation, i.e., methylation occurring on only one DNA strand of a CpG site but not the other. Because demethylation and de novo methylation are related to the loss and gain of methylation respectively, hemimethylation may be associated with the changes of methylation patterns and levels as cancer progresses [7]. That is, hemimethylation may be closely related to abnormal hypermethylation and hypomethylation patterns found in a cancer genome. In fact, Ehrlich and Lacey find that the study of hemimethylation provides valuable insight into cancer-associated active demethylation and hypomethylation [5]. Exactly how different hemimethylation patterns affect gene expression is not yet well documented, except for the recent findings by Xu and Corces, who show that the elimination of hemimethylation can reduce chromatic interactions [8]. They also show that in inner cell mass cells, there are a large number of hemimethylated CpG sites on gene bodies. These hemimethylated sites play a pleiotropic role on gene expression [8].

DNA methylation patterns and levels can vary as cancer progresses [7]. Abnormal hypermethylation and hypomethylation are commonly known characteristics of cancerous cells. Identification of these different states of methylation can assist in the detection of cancerous cells long before they would appear in clinical examinations or before symptoms are apparent. Hemimethylation as a transitional state or indicator of hypomethylation and hypermethylation can help medical researchers to monitor how far the disease has progressed. Knowledge of the hemimethylation can allow for better comprehension of certain cancers and provide better insight toward the development of treatment methods. Therefore, it is important to investigate it in cancer. Hemimethylation has been researched previously for breast cancer cell lines [9], but not yet for lung cancer. Lung cancer is a great candidate for this investigation as it is challenging to detect early-stage lung cancer. Its symptoms are often obscure or mistakable due to the consequence of previous smoking habits. Utilizing hemimethylated genes to identify carcinogenic development may be beneficial in lung cancer diagnosis. The purpose of this research study is to identify hemimethylation patterns in both normal and tumorous samples of non-small cell lung cancer patients using publicly available methylation sequencing data.

## Methods

In this study, to identify hemimethylation patterns, we will analyze the methylation sequencing data generated from tumor and adjacent normal tissues of 18 male non-small cell lung cancer patients in their fifties to seventies. The reduced representation bisulfite sequencing (RRBS) datasets of these patients are publicly available [10]. Sequencing reads are aligned to the hg38 reference genome, and methylation signals are obtained using the BRAT-bw software package [11]. All methylation datasets consist of the methylation signals of CpG sites. These methylation levels are calculated based on the original or raw number of reads, that is, the methylation level or ratio at each CpG site is calculated as the number of methylated reads divided by total number of reads. Further analysis is then performed on CpG sites with at least four methylation signals for both strands.

**Fig. 1:**
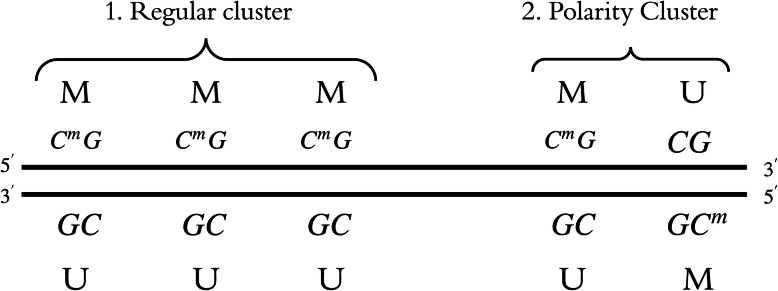
Examples of regular and polarity clusters shown on forward and reverse strands. C^m^G (or GC^m^) refers to a methylated (M) site; CG (or GC) refers to an unmethylated (U) site

Hemimethylation is a particular kind of methylation pattern. If a CpG is methylated on the forward strand but not on the reverse strand, it is defined as a MU hemimethylation site. If a CpG is methylated on the reverse strand but not on the forward strand, it is defined as a UM hemimethylation site. If a CpG exhibits no significant hemimethylation, it is defined as an NS site. If no data is available to be analyzed at a CpG, that site is defined as an NA site. Hemimethylation occurs not only at solitary CpG sites, but also at consecutive ones, known as hemimethylation clusters. Such clusters can manifest in one of two distinct patterns: regular or polar [5, 7, 9, 12], see Fig. 1. A regular cluster can be observed when sequential CpG sites are methylated on the same strand but unmethylated on the other. A polar or polarity cluster occurs when consecutive CpG sites are methylated on opposite strands. Next, we will explain in detail how to identify these different hemimethylation patterns.

The Wilcoxon signed rank test is utilized to investigate if hemimethylation exists at each CpG site [9, 13]. This particular test is selected instead of a regular statistical t-test because the independence and normality assumptions are not satisfied. The null hypothesis is that, at each CpG site, there is no methylation level difference between the forward (or positive) and reverse (or negative) strands. For every CpG site, there are two methylation signals, one from the forward and one from the reverse strand. That is, there are up to 18 pairs of methylation signals at each CpG site as there are 18 samples. For each pair, the forward strand methylation level is subtracted from the reverse strand methylation level. The absolute value of the difference and the sign of the difference (negative or positive) are recorded separately. Pairs with zero difference are discarded. The absolute differences of the pairs are then ranked from smallest to largest so that the pair with the smallest absolute difference is ranked one. Lastly, a test statistic is calculated by summing all of the ranks after multiplying them by their respective signs (i.e., signed-ranks). This test statistic follows a specific distribution, and it is evaluated using a reference table. If the test statistic falls into the rejection region that is determined by the critical value from the table, then the null hypothesis is rejected. The rejection decision means that there is difference between the forward and reverse methylation levels at a CpG site. If the null hypothesis is not rejected, it shows that there is not a significant difference. The Wilcoxon signed rank test is conducted for tumor and normal samples separately. That is, we identify hemimethylated CpG sites for tumor and normal samples separately and then compare them.

The test results are filtered based on two metrics: forward and reverse strand methylation mean difference and Wilcoxon test *p*-value. CpG sites with a large mean difference in methylation levels and a *p*-value that is less than 0.05 are identified as hemimethylated CpG sites. Significant CpG sites are annotated to show which gene promoter region or gene body they belong to. Additionally, clusters of CpG sites are identified as either regular or polar patterns. For example, MMM-UUU and MU-UM are regular and polar clusters respectively, see Fig. 1. MMM-UUU means that at three consecutive CpG sites, methylation occurs on the positive strands (i.e., MMM) but not on the reverse strand (i.e., UUU). MU-UM means at two consecutive CpG sites, on the positive strand they are methylated (M) and unmethylated (U) respectively (i.e., MU), and on the reverse strand they are unmethylated (U) and methylated (M) respectively (i.e., UM). The CpG sites that are not in clusters are called singletons. The lengths of all clusters in tumor and normal cells are shown in histograms. The percentages of CpG sites in regular clusters, polar clusters, and singleton points are summarized. All these summarized results of tumor and normal samples are further compared using statistical tests. For those CpG sites in clusters, DNA strands in the tumor and adjacent normal cells are compared, and overlapping clusters are identified. We’ll show the key findings in the Results section.

## Results

Hemimethylated CpG sites for both normal and tumor cells are identified using the Wilcoxon tests. Table 1 describes the proportions of hemimethylation sites that are in clusters based on the *p*-value (*p* < 0.05) and three mean difference cutoff values. There are similar numbers of hemimethylation sites in tumor and normal samples, but the proportion in clusters is slightly higher in normal samples. When comparing the proportions between normal and tumor, we get the following three *p*-values, 0.00039, 0.00035, and 0.277. These *p*-values correspond to the three mean difference cutoff values 0.4, 0.6, and 0.8 respectively. For the rest of this paper, our analysis will focus on the hemimethylation sites identified based on the *p*-value of 0.05 and the absolute mean difference greater than or equal to 0.4.

**Table 1:** Number of hemimethylated CpG sites and percentage of sites in clusters

|Mean difference|	Normal			Tumor		
	Total	Sites in clusters	Percentage	Total	Sites in clusters	Percentage
≥0.4	7351	1510	20.54%	7330	1336	18.23%
≥0.6	2588	348	13.45%	2743	282	10.28%
≥0.8	723	53	7.33%	823	49	5.95%

Each row is for a mean difference level. The two panels (three columns each) are for normal and tumor samples respectively

Tumor and normal samples’ hemimethylation CpG sites are compared in Table 2. The first row of this table, i.e., the T.MU row, indicates the total number of MU hemimethylation CpG sites in tumor (T) cells. Among these sites, 1697 of them are also hemimethylated in normal cells (N.MU), 1688 of them are not significantly hemimethylated in normal (N.NS), and 217 of them have no data in normal cells (N.NA). The first column of Table 2, i.e., the N.MU column, shows the total number of MU hemimethylation CpG sites in normal (N) cells. Among these sites, 1697 of them are also hemimethylated in tumor cells (T.MU), 1728 of them are not significantly hemimethylated in tumor (T.NS), and 268 of them have no data in tumor cells (T.NA).

**Table 2:** Comparison of normal and tumorous hemimethylation site patterns

	N.MU	N.UM	N.NS	N.NA
T.MU	1697	0	1688	217
T.UM	0	1597	1892	239
T.NS	1728	1789	1,895,429	101,322
T.NA	268	272	98,209	27,295,013

Each row is for the tumor (T) sample and each column is for the normal (N) sample with various hemimethylation types. T.MU refers to CpG sites that are methylated (M) on the forward strand and unmethylated (U) on the reverse strand in tumor (T) samples. N.MU refers to CpG sites with the MU hemimethylation in normal (N) samples. T.NS and N.NS refer to CpG sites of a corresponding tissue type that are not significantly hemimethylated. Similarly, T.NA and N.NA refer to CpG sites that have no data for the given cell type

**Fig. 2:**
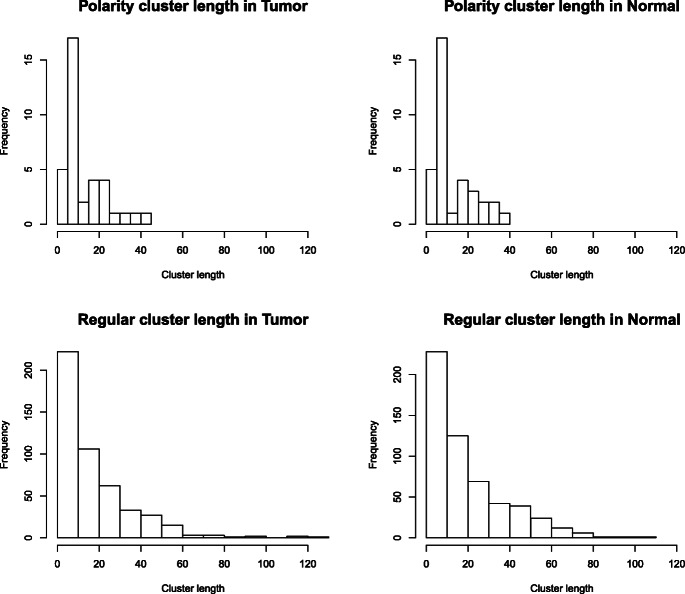
Length of clusters for both normal and tumor samples

Tumor and normal samples’ hemimethylation clusters are compared in Table 3. This table shows that most clusters only have two or three CpG sites and cluster frequency decreases with increased cluster length, meaning large congregations of hemimethylation are infrequent. The length of a cluster is defined as the total number of base pairs between the first and the last CpG sites in the cluster. Figure 2 shows four histograms of cluster lengths. These histograms display the length distributions of polarity patterns in tumor, polarity patterns in normal, regular patterns in tumor, and regular patterns in normal samples. Regular and polarity patterns are analyzed separately because polarity clusters tend to be much shorter. In fact, many of the polarity clusters are less than 40 base pairs long, and a majority of them are less than 10 base pairs long (see peaks in the top panels of Fig. 2). Many of the regular clusters are relatively short, i.e., less than 60 base pairs long, but a small amount of them are longer than that with a maximum length of around 100 to 120 base pairs. A Wilcoxon rank-sum test is performed to compare the difference between the lengths of clusters in normal and tumor cells. The test result is insignificant (*p*-value = 0.12).

**Table 3:** Normal and tumor hemimethylation cluster patterns

Cluster Pattern	Normal	Tumor
MMMMMMMMMMMM-UUUUUUUUUUUU	1	1
MMMMMMMMMM-UUUUUUUUUU	1	1
MMMMMMMM-UUUUUUUU	2	2
MMMMMMM-UUUUUUU	2	2
MMMMMM-UUUUUU	5	3
MMMMM-UUUUU	6	7
MMMM-UUUU	18	13
MMM-UUU	55	32
MM-UU	168	153
MMU-UUM	0	1
MU-UM	28	32
UMM-MUU	1	0
UM-MU	7	4
UUM-MMU	1	0
UU-MM	195	172
UUU-MMM	52	44
UUUU-MMMM	22	22
UUUUU-MMMMM	9	14
UUUUUU-MMMMMM	3	4
UUUUUUM-MMMMMMU	0	1
UUUUUUU-MMMMMMM	4	3
UUUUUUUM-MMMMMMMU	1	0
UUUUUUUU-MMMMMMMM	2	2
Total	583	513

The first column is the cluster pattern, separating forward and reverse strands by “-”. The second and third columns are the counts of such patterns in normal and tumor samples respectively

For the two main hemimethylation cluster patterns, regular cluster and polarity cluster, we summarize them in detail in Tables 4 and 5. Table 4 describes the proportions of different regular clusters in normal and tumor DNA. Table 5 describes the proportions of different polarity patterns in normal and tumor DNA. Polarity clusters appear less frequently than regular patterns, as seen by the difference in the number of sites between Tables 4 and 5. For example, tumor samples have a total of 477 regular clusters and only 36 polar clusters.

**Table 4:** Regular clusters with corresponding percentages

Regular Clusters	Normal		Tumor	
MM-UU	168	30.66%	153	32.075%
UU-MM	195	35.58%	172	36.059%
Bigger cluster	185	33.76%	152	31.866%
Total	548	100%	477	100%

Bigger clusters (see the fourth row) are the ones with 3 or more hemimethylated CpG sites

**Table 5:** Polarity clusters with corresponding percentages

Polarity Clusters	Normal		Tumor	
MU-UM	28	80%	32	88.89%
UM-MU	7	20%	4	11.11%
Total	35	100%	36	100%

**Fig. 3:**
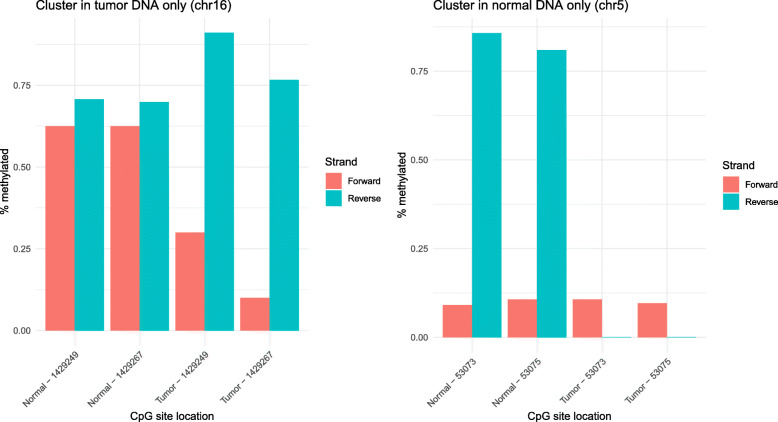
Examples of clusters found in either tumor or normal samples, but not both. The labels beneath each pair of bars describe their exact positions in the genome. The orange red bars represent the percentage of methylation in the forward strand and the cyan blue bars represent the percentage of methylation in the reverse strand. A large disparity between the orange red and cyan blue bars is an indication of hemimethylation

One way to detect which clusters may be related to cancer is to compare the cluster locations between tumor DNA and normal DNA. Some clusters may appear in the same sites in both tumor and normal samples, but others may be found only in tumor or only in normal. In Fig. 3, we show two typical hemimethylation clusters: one that is only identified in tumor DNA and one that is only identified in normal DNA. The first two pairs of bars represent two CpG sites in normal DNA. The second two represent two CpG sites in tumor DNA. We see in the first (or left) plot that there is a large difference between the forward and reverse strands in the tumor CpG sites, whereas the normal CpG sites are quite similar. This tells us that there is a cluster containing two CpG sites that is found only in tumor DNA. Similarly, the second (or right) plot describes a cluster that appears only in normal DNA. In fact, there is almost no methylation in the tumor reverse strands, while the normal reverse strands are almost fully methylated. The forward strand methylation levels are similarly low in tumor and normal DNA, so we observe normal-only hemimethylation in the two sites.

In order to study hemimethylation patterns thoroughly, we compare the 513 tumor clusters with the 583 normal clusters and summarize the results in Table 6. This table shows that multiple kinds of overlaps can be found between tumor and normal. Hemimethylation clusters that occur only in tumor or normal samples are shown in Column B. 695 (313 tumor only and 382 normal only) clusters fall into these categories, and these are the clusters or regions that may be associated with cancer. Column C counts the number of clusters that are exactly the same for normal and tumor. Column D indicates the situations in which a tumor cluster begins and ends within a normal cluster (i.e., a tumor cluster contained within the bounds of a normal cluster), and vice versa as shown in Column E. For example, a tumor cluster’s start and end positions on a chromosome are 150 and 170 base pairs. It is located within a normal cluster that has the start and end positions of 120 and 190 base pairs. Column D, which represents tumor clusters that are embedded in normal clusters, shows different counts for normal and tumor samples because there are two instances of multiple normal clusters located in one tumor cluster. Similarly, Column E, which represents normal clusters that are embedded in tumor clusters, shows different counts because there are three tumor clusters that are located in one normal cluster. Column F represents all other kinds of overlap. For example, there are two normal clusters that have some overlap with the same tumor cluster.

**Table 6:** Tumor and normal cluster comparison results

A	B	C	D	E	F
Tumor Total	Tumor Only	Exact Overlap	Tumor in Normal	Normal in Tumor	Other Overlap
513	313	140	25	23	12
Normal Total	Normal Only	Exact Overlap	Tumor in Normal	Normal in Tumor	Other Overlap
583	382	140	23	25	13

Columns are for different overlap (or non-overlap) patterns. The two rows are for tumor and normal, respectively

The tumor data row of Table 6 shows that among the 513 tumor clusters, 313 of them belong to tumor only; 140 clusters also show up in normal samples; 25 tumor clusters are short ones and they are located within long normal clusters; 23 tumor clusters are long ones in which short normal clusters are located; and 12 tumor clusters are partially overlapped with normal clusters. The normal data row of Table 6 shows that among the 583 normal clusters, 382 of them belong to normal only; 140 clusters also show up in tumor samples; 23 normal clusters are long ones and they cover short tumor clusters; 25 normal clusters are short ones and they are located within long tumor clusters; and 13 normal clusters are partially overlapped with tumor clusters. A detailed version of Table 6 is shown in the Supplemental Table 1 of the Additional File 1, in which the number of different clusters in each chromosome is listed for both tumor and normal samples.

After identifying hemimethylated CpG sites, we may also map them back to genes. That is, we provide the annotation for each CpG site by providing the gene name in whose gene body or promoter region a hemimethylation site is located. We call this analysis gene annotation, and summarizing such will provide the frequency on how many hemimethylated CpG sites a gene has. This annotation analysis is important because highly hemimethylated genes may play an important role. Table 7 shows the frequency of hemimethylated CpG sites in gene bodies. Each column shows how many genes have *n* hemimethylated CpG sites in their gene bodies, where *n* is given in the first row. The second row describes the distribution for tumor genes and the third row describes the distribution for normal genes. Similarly, Table 8 describes the frequency of hemimethylated CpG sites in promoter regions. Table 7 displays that the large majority of gene bodies have at most three hemimethylated CpG sites in both tumor and normal samples, but a few have more than 10. When looking at promoter regions, Table 8 shows none have 10 or more and the large majority of genes have one or two hemimethylated CpG sites.

**Table 7:** Hemimethylation frequency measured in gene bodies for both tumor and normal samples

No. of Hemimethylation sites per gene body (*n*)	1	2	3	4	5	6	7	8	9	≥10
Tumor	1133	250	79	37	17	4	7	2	0	4
Normal	1118	229	73	32	11	4	3	1	1	5

**Table 8:** Hemimethylation frequency measured in promoter regions for both tumor and normal samples

No. of Hemimethylation sites per promoter region (*n*)	1	2	3	4	5	6	7	8
Tumor	223	23	5	6	0	2	0	1
Normal	256	36	13	3	2	1	1	0

With the gene annotation analysis, we can identify genes that have relatively more hemimethylation sites. In particular, we select the genes that have at least five hemimethylation sites in tumor only, in normal only, and in both normal and tumor samples. These genes are summarized in Tables 9, 10, and 11 respectively. Note, there are not many genes with a large number of hemimethylated sites. Therefore, we choose a relatively small number (i.e., five) to find a reasonable number of genes that meet this criterion for us to do further analysis. In addition, the datasets used in this project are generalized using the RRBS method. For this method, only a small percent of the CpG sites in a genome are sequenced [12, 16]. If the methylation sequencing datasets used in this study are generated based on the whole genome bisulfite sequencing method, more hemimethylated CpG sites can be found in different genes.

**Table 9:** For genes with at least five hemimethylation sites in tumor samples

Gene name	Count	Family	Gene Description
RGS14	17	–	regulator of G protein signaling 14
MEX3A	16	–	mex-3 RNA binding family member A
WT1	11	TF, TS	WT1 transcription factor
PRDM16	10	OG, TF, TCG	PR/SET domain 16
ZDHHC9	10	–	zinc finger DHHC-type containing 9
AGAP2	8	–	ArfGAP with GTPase domain, ankyrin repeat and PH domain 2
GNAS	8	OG	GNAS complex locus
EXOC3L2	8	–	exocyst complex component 3 like 2
PTPRN2	7	–	protein tyrosine phosphatase receptor type N2
FANK1	7	–	fibronectin type III and ankyrin repeat domains 1
UNC93B1	7	–	unc-93 homolog B1, TLR signaling regulator
IGSF9B	7	–	immunoglobulin superfamily member 9B
GNAS-AS1	7	–	GNAS antisense RNA 1
MAD1L1	7	–	mitotic arrest deficient 1 like 1
TSPAN9	7	–	tetraspanin 9
PTPRM	7	–	protein tyrosine phosphatase receptor type M
TP73	6	TF	tumor protein p73
IFT140	6	–	intraflagellar transport 140
NFATC1	6	TF	nuclear factor of activated T cells 1
DGKA	6	–	diacylglycerol kinase alpha
FMNL1	6	–	formin like 1
CACNA1I	6	–	calcium voltage-gated channel subunit alpha1 I
LOC101927636	6	–	RNA Gene affiliated with the lncRNA class
HDAC4	5	TF	histone deacetylase 4
IRX2	5	TF, HP	iroquois homeobox 2
ANKRD33B	5	–	ankyrin repeat domain 33B
LINC00537	5	–	Long Intergenic Non-Protein Coding RNA 537
NOTCH1	5	OG, TCG	notch receptor 1
ANO2	5	–	anoctamin 2
CACNA1H	5	–	calcium voltage-gated channel subunit alpha1 H
RUNX3	5	TF	runt related transcription factor 3
SIX3	5	TF, HP	SIX homeobox 3
FZD7	5	–	frizzled class receptor 7
ADGRA2	5	–	adhesion G protein-coupled receptor A2
IFFO1	5	–	intermediate filament family orphan 1
CHTF18	5	–	chromosome transmission fidelity factor 18
TMEM204	5	–	transmembrane protein 204
RECQL5	5	–	RecQ like helicase 5
SMIM5	5	–	small integral membrane protein 5
MAPK1	5	PK	mitogen-activated protein kinase 1
SYN1	5	–	synapsin I

The gene name, corresponding number of hemimethylated sites (i.e., count), specified gene family, and a description of the gene are formatted in the table’s respective columns. Descriptions are derived from the Molecular Signature Database [14] and the GeneCards database [15]. Certain genes are indicated as members of specific gene families, as shown in the third column: “TF” for transcription factor, “TS” for tumor suppressor, “OG” for oncogene, “HP” for homeodomain protein, “TCG” for translocated cancer gene, and “PK” for protein kinase

**Table 10:** For genes with at least five hemimethylation sites in normal samples

Gene name	Count	Family	Gene Description
ZFPM1	14	TF	zinc finger protein, FOG family member 1
GNAS	13	OG	GNAS complex locus
RGPD2	12	–	RANBP2 like and GRIP domain containing 2
SHANK3	11	–	SH3 and multiple ankyrin repeat domains 3
IRX2	10	TF, HP	iroquois homeobox 2
LTB4R	9	–	leukotriene B4 receptor
CPEB3	8	–	cytoplasmic polyadenylation element binding protein 3
PTPRN2	7	–	protein tyrosine phosphatase receptor type N2
MIR1268A	7	–	microRNA 1268a
GNAS-AS1	7	–	GNAS antisense RNA 1
CYP26C1	7	–	cytochrome P450 family 26 subfamily C member 1
TBL1XR1	6	–	transducin beta like 1 X-linked receptor 1
HOXA3	6	TF, HP	homeobox A3
CACNA1H	6	–	calcium voltage-gated channel subunit alpha1 H
NPEPPS	6	–	aminopeptidase puromycin sensitive
SEMA6B	6	CGF	semaphorin 6B
HOMER3	6	–	homer scaffold protein 3
PINLYP	6	–	phospholipase A2 inhibitor and LY6/PLAUR domain containing
GDI1	6	–	GDP dissociation inhibitor 1
HS3ST2	6	–	heparan sulfate-glucosamine 3-sulfotransferase 2
PRDM16	5	TF, OG, TCG	PR/SET domain 16
PLK3	5	PK	polo like kinase 3
GREM2	5	CGF	gremlin 2, DAN family BMP antagonist
MEIS1	5	TF, HP	Meis homeobox 1
MEIS1-AS2	5	–	MEIS1 antisense RNA 2
POLH	5	–	DNA polymerase eta
HOXA-AS2	5	–	HOXA cluster antisense RNA 2
EBF3	5	–	EBF transcription factor 3
CBFA2T3	5	TF, OG, TCG	CBFA2/RUNX1 translocation partner 3
RPL13	5	–	ribosomal protein L13
NFIC	5	TF	nuclear factor I C
CDH4	5	–	cadherin 4
PDGFB	5	OG, TCG	cytokine or growth factor, platelet derived growth factor subunit B
CCNT1	5	–	cyclin T1
SNORD68	5	–	small nucleolar RNA, C/D box 68

The gene name, corresponding number of hemimethylated sites (i.e., count), specified gene family, and a description of the gene are formatted in the table’s respective columns. Descriptions are derived from the Molecular Signature Database [14] and the GeneCards database [15]. Certain genes are indicated as members of specific gene families, as shown in the third column: “TF” for transcription factor, “TS” for tumor suppressor, “OG” for oncogene, “HP” for homeodomain protein, “TCG” for translocated cancer gene, and “PK” for protein kinase

**Table 11:** For genes with at least five hemimethylation sites in both tumor and normal samples

Gene name	Count	Family	Gene Description
RGPD5	16	–	RANBP2 like and GRIP domain containing 5
RGPD8	16	–	RANBP2 like and GRIP domain containing 8
ROCK1P1	13	–	Rho associated coiled-coil containing protein kinase 1 pseudogene 1
THAP4	8	–	THAP domain containing 4
SGTA	8	–	small glutamine rich tetratricopeptide repeat containing alpha
PTPRN2	7	–	protein tyrosine phosphatase receptor type N2
CNTNAP3	7	–	contactin associated protein like 3
NUTM2A-AS1	7	–	NUTM2A antisense RNA 1
RBFOX3	7	–	RNA binding fox-1 homolog 3
ESPNP	6	–	espin pseudogene
FOXK1	6	–	forkhead box K1
HOXA3	6	HP, TF	homeobox A3
LMF1	6	–	lipase maturation factor 1
USP45	6	–	ubiquitin specific peptidase 45
LOC101928782	6	–	RNA Gene affiliated with the lncRNA class
PRDM16	5	OG, TF, TCG	PR/SET domain 16
RGPD4	5	–	RANBP2 like and GRIP domain containing 4
MERTK	5	PK	MER proto-oncogene, tyrosine kinase
FAM160A1	5	–	family with sequence similarity 160 member A1
PRKAR1B	5	–	protein kinase cAMP-dependent type I regulatory subunit beta
MAD1L1	5	–	mitotic arrest deficient 1 like 1
HOXA2	5	HP, TF	homeobox A2
DPP6	5	–	dipeptidyl peptidase like 6
DIP2C	5	–	disco interacting protein 2 homolog C
FANK1	5	–	fibronectin type III and ankyrin repeat domains 1
GAL3ST3	5	–	galactose-3-O-sulfotransferase 3
FLJ12825	5	–	RNA Gene affiliated with the lncRNA class
KLF5	5	TF	Kruppel like factor 5
ISL2	5	HP, TF	ISL LIM homeobox 2
CBFA2T3	5	OG, TF, TCG	CBFA2/RUNX1 translocation partner 3
SBNO2	5	–	strawberry notch homolog 2
GIPR	5	–	gastric inhibitory polypeptide receptor
SCAF1	5	–	SR-related CTD associated factor 1
COL6A1	5	–	collagen type VI alpha 1 chain
NEXMIF	5	–	neurite extension and migration factor
GK5	5	–	glycerol kinase 5

The gene name, corresponding number of hemimethylated sites (i.e., count), specified gene family, and a description of the gene are formatted in the table’s respective columns. Descriptions are derived from the Molecular Signature Database [14] and the GeneCards database [15]. Certain genes are indicated as members of specific gene families, as shown in the third column: “TF” for transcription factor, “TS” for tumor suppressor, “OG” for oncogene, “HP” for homeodomain protein, “TCG” for translocated cancer gene, and “PK” for protein kinase

There are 41 genes with the most hemimethylation in tumor DNA, see Table 9. Among these genes, TP73 [17–19], GNAS [20–24], and NOTCH1 [25, 26] are notable ones with known relations to cancer. Table 9 shows that among these 41 genes, one is a tumor suppressor (WT1), three are oncogenes (GNAS, NOTCH1, and PRDM16), and of those three, two are translocated cancer genes (NOTCH1 and PRDM16). There are also eight transcription factors in this table (HDAC4, IRX2, NFATC1, PRDM16, RUNX3, SIX3, TP73, and WT1). Table 10 shows 35 genes with the most hemimethylation in normal DNA. Among these genes, four are oncogenes (CBFA2T3, GNAS, PDGFB and PRDM16). Of the oncogenes, three are translocated cancer genes (CBFA2T3, PDGFB and PRDM16). There are also seven transcription factors in this table (CBFA2T3, HOXA3, IRX2, MEIS1, NFIC, PRDM16, and ZFPM1). Note that no tumor suppressor genes are hemimethylated in the normal cells. For genes belonging to two key gene families (i.e., transcription factor and oncogene), we have compared their proportions in tumor and normal samples using statistical tests. The test *p*-values are 0.96 for the transcript factor family and 0.54 for the oncogene family. There is no significant difference. Table 11 shows 36 genes with the most hemimethylation in both normal and tumor DNA. Among these genes, two are oncogenes and also translocated cancer genes (CBFA2T3 and PRDM16). There are also six transcription factors in this table (KLF5, HOXA2, CBFA2T3, HOXA3, ISL2, and PRDM16). All three gene tables have some transcription factor genes, which may affect the gene expression of other cancer-related genes that are not found to be hemimethylated.

**Fig. 4:**
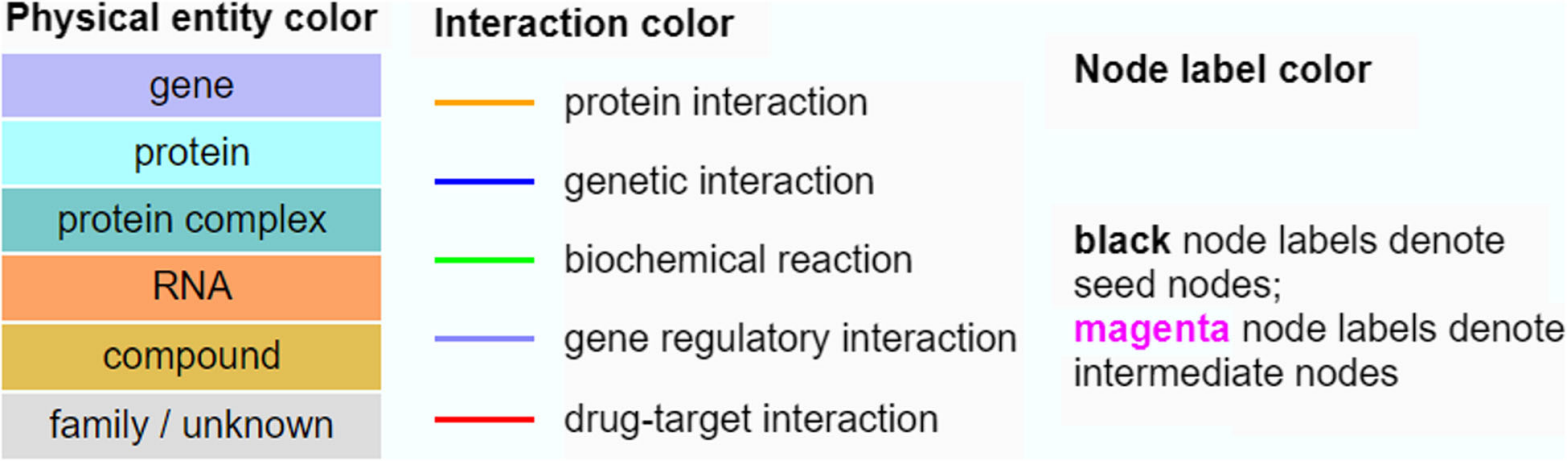
Key for gene relationship diagrams in Figs. 5, 6, and 7

**Fig. 5:**
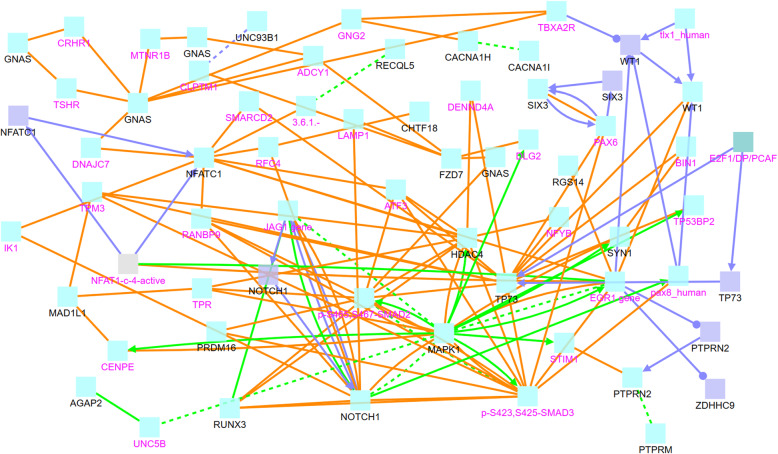
Relationship between genes with ≥5 hemimethylation sites in tumor samples

**Fig. 6:**
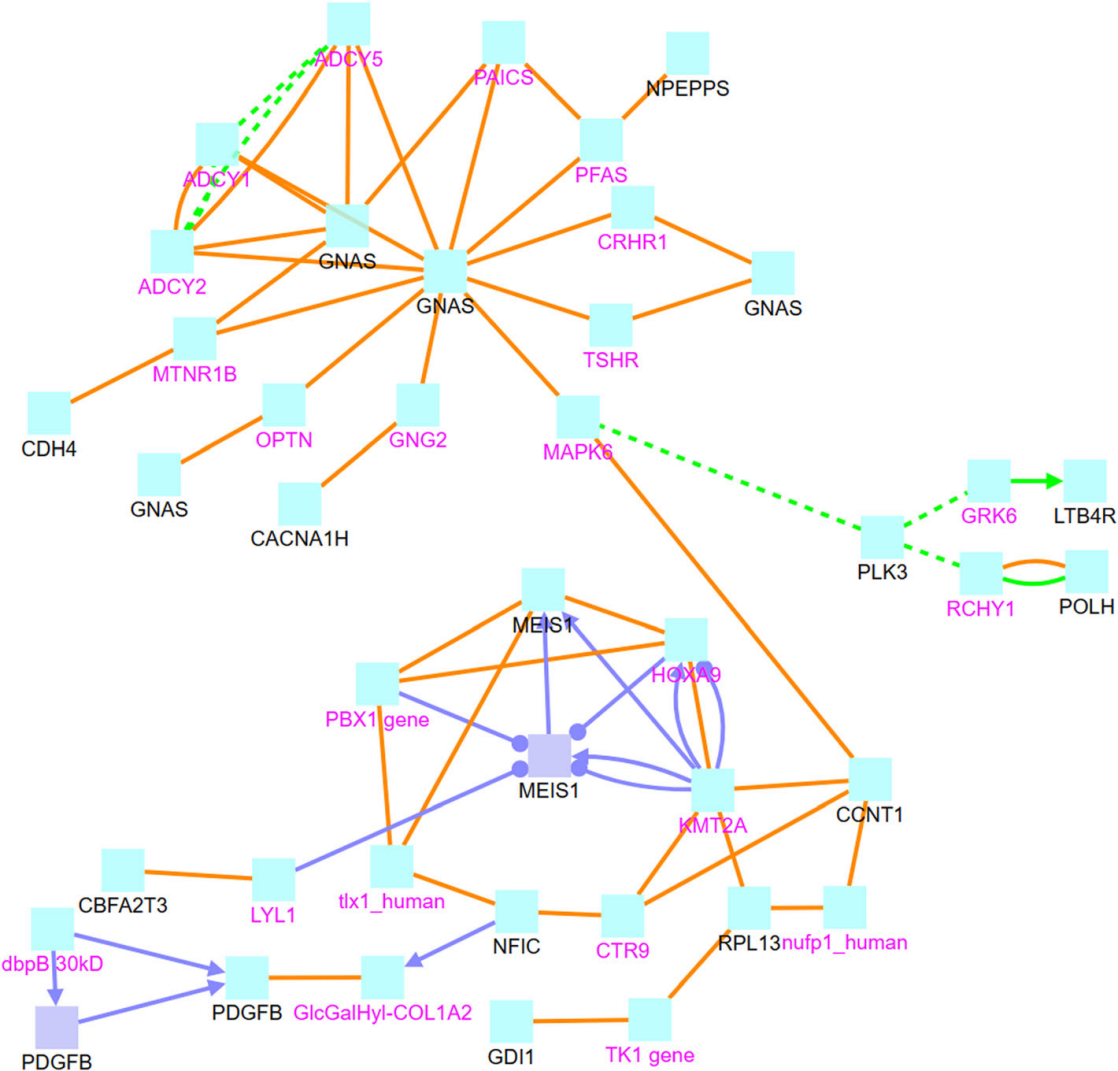
Relationship between genes with ≥5 Hemimethylation sites in normal samples

**Fig. 7:**
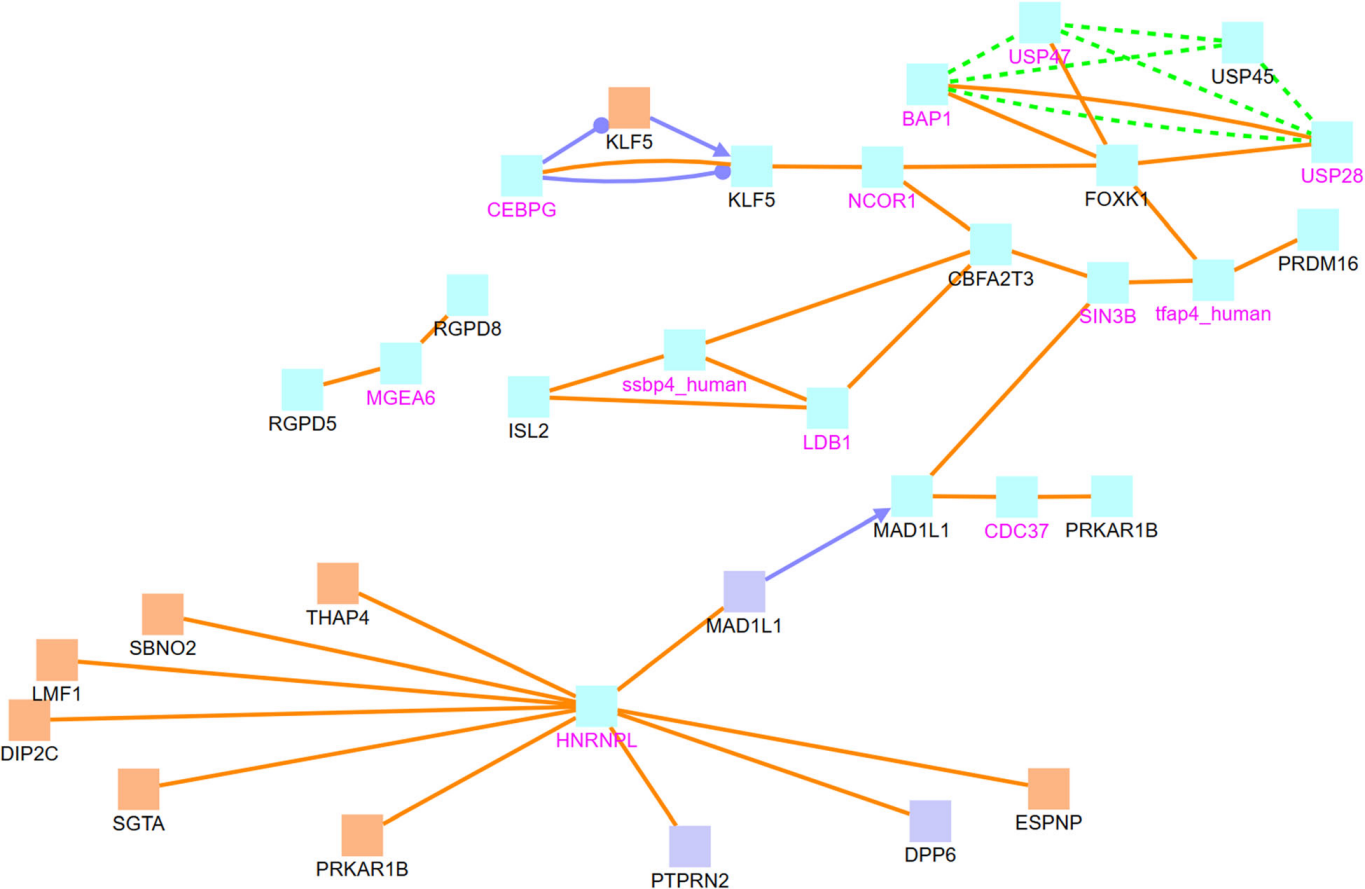
Relationship between genes with ≥5 hemimethylation sites identical in both tumor and normal samples

In order to understand the functions and relationships of these genes, we further analyze their biological interactions using the ConsensusPath Database (CPDB) software package [27–29], see Figs. 4, 5, 6, and 7. Figure 4 describes the different types of biological relationships between genes based on the CPDB software. A gene with a black label is known to be hemimethylated (i.e., identified by our analysis). A gene with a purple label is not provided in our hemimethylation gene list but interacts with one of the known genes. Figure 4 is the legend for Figs. 5, 6, and 7. This legend figure summarizes the relationships for gene lists in Tables 9, 10, and 11 as shown in Figs. 5, 6, and 7, respectively. These figures show the extent to which these highly hemimethylated genes interact and possibly affect the cell function of related genes.

Figure 5 shows genetic interactions between genes with the most hemimethylation in tumor samples, and these genes are recorded in Table 9. The gene network in Fig. 5 contains a number of hub genes with complex interactions. These hub genes include GNAS, NFATC1, NOTCH1, MAPK1, HOAC4, TP73, and EGR1. We can see that if a hub gene like MAPK1 is hemimethylated, it may interact with dozens of other genes. Some of these genes, e.g., EGR1 [30–33] and UNC5B [34–37], are known to be associated with different cancers, including lung cancer. EGR1 has a promoting effect on cancer metastasis in OCT4-overexpressing lung cancer [38]. The pseudogene DUXAP8 may act as an oncogene in non-small cell lung cancer, and it may play this role by silencing EGR1 and RHOB transcription via binding with EZH2 and LSD1 [39]. The expression of UNC5A, UNC5B, or UNC5C is down-regulated in multiple cancers including lung cancer [40], and UNC5B has also been indicated as a putative tumor suppressor [41].

Figure 6 shows genetic interactions between genes with the most hemimethylation in normal DNA, and these genes are recorded in Table 10. In this figure, we can see that GNAS is a hub gene interacting with many other genes that may not be hemimethylated themselves. GNAS is observed in both tumor and normal samples, as well as in the hemimethylation study for breast cancer cell lines [9]. MEIS1 is also a hub gene that interacts with genes like KMT2A [42] and TK1 [43]. While these genes are not hemimethylated in our samples, they are known to be associated with cancer. KMT2A and hTERT are positively correlated in melanoma tumor tissues, and KMT2A promotes melanoma cell growth by targeting the hTERT signaling pathway [44]. KMT2A has an epigenetic regulation role on NOTCH1 and NOTCH3, and this mechanism is essential for inhibiting glioma proliferation [45]. TK1 plays a moderate role as a diagnostic tumor marker for cancer patients [46], and it is a potential clinical biomarker for the treatment of lung, breast, and colorectal cancer [47]. A systematic review shows that TK1 overexpression is associated with the poor outcomes of lung cancer patients [48]. MEIS1 inhibits non-small cell lung cancer cell proliferation [49]**.** MEIS1 plays a crucial role in normal development [15] and it is also reported as an important gene related to leukemia [50–52]. Therefore, it is possible that the hemimethylation of hub genes like MEIS1 affects protein, biochemical, or regulatory functions of genes that are associated with cancer.

Figure 7 shows genetic interactions between genes with the most hemimethylation on identical locations in tumor and normal samples. These genes are recorded in Table 11. This means that the hemimethylation of CpG sites in this network is unchanged or unaffected by the formation of cancer. The HNRNPL gene is a major hub in this gene network. While we do not detect any hemimethylation in this gene, it directly interacts with 10 genes that we know to be hemimethylated. Some of these genes, like PTPRN2 and MAD1L1, can also be found in the tumor gene network, see Fig. 5. There appears to be no common genes between Fig. 6 (hemimethylated genes in normal samples) and Fig. 7 (hemimethylated genes in both tumor and normal samples). Therefore, genes that have a large number of hemimethylated CpG sites found only in normal DNA seem to have few CpG sites that remain the same when cancer forms.

In addition to the above analysis, we have conducted gene set enrichment analysis using the molecular signature database and the related software package provided by the Broad Institute [14]. Of the most hemimethylated genes in tumor DNA (Table 9), six are also significantly represented in cancer module 163 (with *p*-value < 0.05). This module is a collection of genes known to be overrepresented in cancer pathways and is reported by the Stanford research group (http://robotics.stanford.edu/~erans/cancer/modules/). The six genes are IFT140, IFFO1, SYN1, FMNL1, NOTCH1, and RGS14. There are no such overly represented genes and cancer modules among genes shown in Table 10 (for normal samples) and Table 11 (for both tumor and normal samples).

## Discussion

It was previously believed that hemimethylation appears only in a transient state [4]. However, Shao et al. have reported hemimethylated sites and patterns in ovarian cancer [7]. Sun et al. have identified hemimethylation patterns in breast cancer cell lines [9]. Furthermore, Xu and Corces have shown that some hemimethylation sites can be inherited across cell divisions. They have also shown that hemimethylated CpG sites account for 4–20% of the DNA methylome in different cell types [53]. Therefore, hemimethylation may serve as a stable epigenetic mark. In addition, recent papers show that hemimethylation is a characteristic of secondary differential methylation regions that are associated with imprinted genes [54–56]. That is, hemimethylation is a novel epigenetic modification functional for genomic imprinting. All these recent findings challenge the previous prevailing view of hemimethylation. It is unlikely that all hemimethylation sites in a genome are transient; instead, certain hemimethylation sites or patterns may have a stable and important impact on the overall methylation.

DNA methylation is closely related to other genetic events or patterns, e.g., mutation. There is a significantly positive correlation between differential methylation and tumor mutation burden (i.e., the frequency of certain mutations) as shown in a recent non-small cell lung cancer study [57]. Differential methylation sites are also identified between T53 mutated and T53 wild type tumors [58]. In addition, we have compared our hemimethylated genes in Tables 9 and 10 with mutated genes obtained from publicly available databases. When comparing with mutated cancer driver genes from the Integrative Onco Genomics [59], we find some of these genes in our Tables 9 and 10. In particular, four tumor-only genes from our Table 9 (NOTCH1, GNAS, MAPK1, WT1) and five normal-only genes from our Table 10 (GNAS, TBL1XR1, CPEB3, NPEPPS, CBFA2T3) are in this cancer driver gene list. Thus, about 10% of our hemimethylated genes are also mutated cancer driver genes. When comparing with the lung cancer mutation genes obtained from the database DriverDBv3 [60], we find that 13 tumor-only genes (in Table 9) and 10 normal-only genes (in Table 10) are in this gene list. That is, about 1/3 of our top hemimethylated genes are lung cancer mutation genes. Note, the 13 tumor-only genes from Table 9 are FMNL1, RGS14, WT1, IFT140, CACNA1H, AGAP2, CACNA1I, ADGRA2, SYN1, GNAS, NFATC1, PRDM16, and MAD1L1. The 10 normal-only genes from Table 10 are CACNA1H, RGPD2, SEMA6B, CYP26C1, GNAS, EBF3, PRDM16, CDH4, MEIS1, and TBL1XR1. The above findings show that methylation and mutation are closely related. It is likely that hemimethylation and mutation are associated as well.

The mean difference cutoff values are 0.4, 0.6 and 0.8 as used in a previous research [9]. Results are narrowed down to the 0.4 cutoff level to allow more results to be viewed, as the higher cutoff values restricted the available hemimethylated CpG sites from being identified. The number of both tumor and normal clusters detected decreases rapidly as we increase the mean cutoff value at each CpG site as shown in Table 2. With more strict criteria, the methylation difference between the two DNA strands at each CpG site must exist in order for us to consider hemimethylation at a CpG site. This rapid decrease may indicate certain hemimethylation heterogeneity in lung cancer as cancer methylation patterns are generally heterogeneous among multiple patients or cell lines [61].

For the 41 most hemimethylated genes in lung cancer tumors, seven of them are also highly hemimethylated in breast cancer cell lines, as reported by Sun et al. [9]. These seven genes are PRDM16, GNAS, PTPRN2, MAD1L1, HDAC4, NOTCH1, and CACNA1H. The remaining 34 highly hemimethylated genes in the lung tumor sample are not highly hemimethylated in breast cancer cell lines. It is possible that these genes are unique to lung cancer; thus, it would be helpful when diagnosing patients with lung cancer specifically, but further research needs to be done.

Based upon the outcome of hemimethylation research in breast cancer cell lines, the frequency of polarity clusters is much higher than the one in this paper. The results of breast cancer hemimethylation analysis indicate polarity clusters are more frequently found than regular clusters [9]. However, the lung cancer analysis reflects contrasting results; polarity clusters are less frequently found than regular clusters for both tumor and normal samples, as shown in Fig. 2, Tables 4 and 5. A couple of factors may explain this difference. One factor could be the type of cancer, as hemimethylation frequency may be tissue specific. Another factor could be that the previous breast cancer study is performed using cell lines, which are tumors grown in labs over a long period of time; whereas, our current study uses primary tissues directly from lung cancer patients. Due to the nature of cell lines and our primary tissues, it is likely that hemimethylation patterns, especially polarity clusters, are related to tumor growth. Polarity clusters are evidence of active demethylation in cancer cells; DNA demethylation is closely related to cancer hypomethylation [7, 62]. Therefore, the identification of polarity clusters in cancer is of direct importance to the study of carcinogenesis. Future research on the pathological significance of polarity clusters in different tumors may reveal more insight into cancer studies.

After conducting statistical tests for a large number of CpG sites, selecting the significant CpG sites is a crucial step, and the multiple testing correction is important because using only the raw *p*-values may result in many false positive sites. However, for the understudied hemimethylation pattern, the proper way of doing multiple testing correction is not clear. In order to explore the impact of different corrections, we have used three methods: a simple moving-average based method, the comb-p FDR method, and the comb-p SLK method. Note, comb-p is a software package developed for combining, analyzing, and correcting spatially correlated *p*-values [63]. FDR stands for the Benjamini–Hochberg false discovery correction [64]. SLK represents the Stouffer–Liptak–Kechris correction [65]. After exploring various correction methods, we conclude that the mean difference plus *p*-value filtering method used in our study can produce meaningful and interpretable results when dealing with the multiple testing comparison problem for our hemimethylation analysis. For detailed comparative analysis results, see the Supplemental Tables 2, 3, and 4 and related explanation in the Additional File 1.

As for the criteria we used to identify hemimethylation sites, in addition to *p*-values and mean differences, we may add an additional one. That is, the methylation signal on one DNA strand is zero, and the methylation signal on the other strand is positive. Adding this criteria will help us to identify hemimethylation sites more strictly with no methylation on one strand but a high methylation signal on another strand. This criterion is ideal when the datasets are not very noisy and when the samples are not heterogeneous. In this project, we choose to only use *p*-value and mean difference for the following reasons. First, we use these two criteria to make a fair comparison between the previous breast cancer results [9] and our new lung cancer results. Second, the average methylation signals at most CpG sites tend to be clustered around 0 or 1 [66]. Third, bisulfite converted methylation sequencing data can be noisy, and tumor samples’ methylation signals are very heterogeneous. We generally consider that the average methylation signals around 0 to 0.2 (or 0.25) are still roughly in the category of no or very low methylation signals [61]. Therefore, most of the CpG sites we identified still tend to have relatively high methylation signals on one DNA strand and have relatively low or no methylation signals on another strand.

## Conclusion

Hemimethylation is an important but understudied pattern in cancer. In this paper, we have conducted the first-ever exploratory investigation of hemimethylation in lung cancer. In particular, we have conducted statistical analyses to identify hemimethylation patterns for non-small cell lung cancer patients. We have identified both singleton hemimethylation sites and different clusters in normal and tumor cells. We have also conducted bioinformatic analysis on the genes that have relatively more hemimethylated sites in tumor, normal, and both tumor and normal cells to see the biological interactions of these genes. Our results show that not only does hemimethylation exist in lung cells, but also with diverse patterns and frequencies that are comparable between normal and tumorous cells. We conclude that hemimethylation is related to both normal and tumor cell development. This is also seen by its existence in the same genes in normal and lung tumor cells. However, there are certain genes that only have hemimethylated sites for one type of cell, normal or tumor, but not both. Certain genes are previously known to be associated with carcinogenesis. These genes exhibit existence in one cell type and not the other. Hemimethylation existing in this way may imply epigenetic changes in certain genes associated with lung cancer. The development and progression of lung cancer may be tracked by the analysis of epigenetic change (i.e., hemimethylation and methylation) in these regions.

## Supplementary Information

**Additional file 1.** This file includes two sections. Section 1: Tumor and normal cluster comparison results by chromosome (Supplemental Table 1). Section 2: Comparative analysis of three methods of multiple testing corrections (Supplemental Tables 2–4)

## Data Availability

Datasets analyzed for this study are publicly available (SRP125064) and can be downloaded from this web page: https://www.ncbi.nlm.nih.gov/sra/SRP125064. All datasets supporting our findings are presented within the manuscript and the additional supporting file.

## Abbreviations

CpG: The shorthand notation for 5′-cytosine-phosphate-guanine-3′
MU: When it is for one CpG site on two DNA strands, it refers to a hemimethylated CpG site with methylation (M) on the positive strand and unmethylation (U) on the reverse strand. When it refers to two consecutive CpG sites on one DNA strand, it means that methylation occurs on the first CpG site (i.e., M), but not on the second one (i.e., U)
UM: When it is for one CpG site on two DNA strands, it refers to a hemimethylated CpG site with unmethylation (U) on the positive strand and methylation (M) on the reverse strand. When it refers to two consecutive CpG sites on one DNA strand, it means that methylation does not occur on the first CpG site (i.e., U), but occurs on the second one (i.e., M)
NS: A CpG site identified as not significantly (NS) hemimethylated
RRBS: Reduced representation bisulfite sequencing
