# Supplementary material for: Statistical and bioinformatic analysis of hemimethylation patterns in non-small cell lung cancer
Source: BMC Cancer. 2021 Mar 12;21:268. doi: 10.1186/s12885-021-07990-7 (PMC7953768; doi:10.1186/s12885-021-07990-7)
Supplement: Supplementary file 1 — Additional file 1. This file includes two sections. Section 1: Tumor and normal cluster comparison results by chromosome (Supplemental Table 1). Section 2: Comparative analysis of three methods of multiple testing corrections (Supplemental Tables 2–4) [file 12885_2021_7990_MOESM1_ESM.docx]

**Additional file for the following paper**

**Statistical and Bioinformatic Analysis of Hemimethylation Patterns**

**in non-small cell lung cancer**

**Shuying Sun^1*^, Austin Zane^2^, Carolyn Fulton^3^, and Jasmine Philipoom^4^**

^1^ Department of Mathematics, Texas State University, San Marcos, Texas, USA,

ssun5211@yahoo.com or s_s355@txstate.edu

^2^ Department of Statistics, Texas A&M University, College Station, Texas, USA,

[azane8@tamu.edu](mailto:azane8@tamu.edu)

^3^ Department of Mathematics, Schreiner University, Kerrville, Texas, USA,

[carriefulton00@yahoo.com](mailto:carriefulton00@yahoo.com)

^4^ Department of Mathematics, Applied Mathematics, and Statistics, Case Western Reserve

University, Cleveland, Ohio, USA, [jnp32@case.edu](mailto:jnp32@case.edu)

^*^Corresponding author: Dr. Shuying Sun, [ssun5211@yahoo.com](mailto:ssun@txstate.edu)

**Section 1: Tumor and normal cluster comparison results by chromosome (a stratified version of Table 6 in the main text)**

**Supplemental table 1:** Tumor and normal cluster comparison results by chromosome.

| chr | type | total | only | exact_overlap | T_in_N | N_in_T | other |
| --- | --- | --- | --- | --- | --- | --- | --- |
| chr1 | T | 37 | 25 | 9 | 1 | 1 | 1 |
| chr1 | N | 49 | 36 | 9 | 1 | 1 | 2 |
| chr2 | T | 32 | 18 | 9 | 4 | 1 | 0 |
| chr2 | N | 35 | 21 | 9 | 4 | 1 | 0 |
| chr3 | T | 22 | 10 | 11 | 0 | 1 | 0 |
| chr3 | N | 30 | 18 | 11 | 0 | 1 | 0 |
| chr4 | T | 17 | 9 | 6 | 0 | 1 | 1 |
| chr4 | N | 21 | 13 | 6 | 0 | 1 | 1 |
| chr5 | T | 29 | 26 | 3 | 0 | 0 | 0 |
| chr5 | N | 19 | 16 | 3 | 0 | 0 | 0 |
| chr6 | T | 14 | 8 | 6 | 0 | 0 | 0 |
| chr6 | N | 29 | 23 | 6 | 0 | 0 | 0 |
| chr7 | T | 38 | 14 | 14 | 2 | 6 | 2 |
| chr7 | N | 40 | 16 | 14 | 2 | 6 | 2 |
| chr8 | T | 23 | 17 | 4 | 1 | 1 | 0 |
| chr8 | N | 13 | 7 | 4 | 1 | 1 | 0 |
| chr9 | T | 23 | 16 | 4 | 1 | 0 | 2 |
| chr9 | N | 27 | 20 | 4 | 1 | 0 | 2 |
| chr10 | T | 12 | 7 | 4 | 0 | 1 | 0 |
| chr10 | N | 20 | 15 | 4 | 0 | 1 | 0 |
| chr11 | T | 21 | 17 | 2 | 1 | 0 | 1 |
| chr11 | N | 24 | 20 | 2 | 1 | 0 | 1 |
| chr12 | T | 27 | 18 | 7 | 1 | 0 | 1 |
| chr12 | N | 26 | 17 | 7 | 1 | 0 | 1 |
| chr13 | T | 8 | 3 | 1 | 2 | 2 | 0 |
| chr13 | N | 12 | 6 | 1 | 2 | 3 | 0 |
| chr14 | T | 16 | 9 | 4 | 2 | 1 | 0 |
| chr14 | N | 19 | 12 | 4 | 2 | 1 | 0 |
| chr15 | T | 11 | 6 | 3 | 1 | 1 | 0 |
| chr15 | N | 16 | 10 | 3 | 1 | 2 | 0 |
| chr16 | T | 25 | 14 | 7 | 1 | 2 | 1 |
| chr16 | N | 38 | 27 | 7 | 1 | 2 | 1 |
| chr17 | T | 25 | 16 | 8 | 1 | 0 | 0 |
| chr17 | N | 22 | 13 | 8 | 1 | 0 | 0 |
| chr18 | T | 10 | 7 | 2 | 1 | 0 | 0 |
| chr18 | N | 9 | 6 | 2 | 1 | 0 | 0 |
| chr19 | T | 55 | 33 | 15 | 4 | 2 | 1 |
| chr19 | N | 56 | 36 | 15 | 2 | 2 | 1 |
| chr20 | T | 18 | 11 | 6 | 0 | 1 | 0 |
| chr20 | N | 24 | 17 | 6 | 0 | 1 | 0 |
| chr21 | T | 6 | 4 | 2 | 0 | 0 | 0 |
| chr21 | N | 4 | 2 | 2 | 0 | 0 | 0 |
| chr22 | T | 17 | 7 | 8 | 0 | 1 | 1 |
| chr22 | N | 17 | 7 | 8 | 0 | 1 | 1 |
| chrX | T | 22 | 15 | 3 | 2 | 1 | 1 |
| chrX | N | 31 | 24 | 3 | 2 | 1 | 1 |
| chrY | T | 5 | 3 | 2 | 0 | 0 | 0 |
| chrY | N | 2 | 0 | 2 | 0 | 0 | 0 |

*This supplemental table is a stratified version of the Table 6 in the main text. As for the column names, “chr” means chromosome; “type” means tumor (T) or normal (T); “total “means total number of clusters; “only” means the number of clusters that are identified in tumor or normal only; “exact_overlap” means clusters are identified exactly in both tumor and normal; “T_in_N”, means the tumor cluster is shorter/smaller and is located in a longer/larger normal cluster; “N_in_T”, means the normal cluster is shorter or smaller and is located in a longer/larger tumor cluster; “other” means the clusters are overlapped in a different pattern that is not “exact_overlp”, “T_in_N”, or “N_in_T”.*

**Section 2 Comparative analysis of three multiple testing correction methods**

After conducting statistical tests for a large number of CpG sites, selecting the significant CpG sites is a crucial step, and multiple testing correction is important because using only the raw p-values may result in many false positive sites. However, for the understudied hemimethylation pattern, the proper way of doing multiple testing correction is not clear. In order to explore the impact of different correction methods, we have used the following three methods: a simple moving-average based method, the comb-p FDR method, and the comb-p SLK method. Note, comb-p is a software package developed for combining, analyzing, and correcting spatially correlated p-values [1]. FDR stands for the Benjamini–Hochberg false discovery correction [2]. SLK represents the Stouffer–Liptak–Kechris correction [3].

For the simple moving-average based p-value correction, the p-value for each CpG site is averaged with the p-values of its two neighboring sites within d (d=25, 50, and 100) base pairs on each site of the genome. If either of the neighbors is further than the d base pairs away, they are not counted in the average. The results in the top panel (top 4 columns) of **Supplemental Table 2** show that adjusting the p-values using moving average results in a large decrease in the number of significant CpG sites. However, it still identifies a much larger number of CpG sites than the number of sites selected using both mean difference and p-value as shown in the **Supplemental Table 2** bottom panel. The differences are 36,521 vs 7,351 (for normal) and 33,431 vs. 7,330 (for tumor). When we apply the mean difference filter after adjusting the p-values with the moving average method, the number of significant CpG sites is further reduced to 3,354 in normal and 3,030 in tumor samples. It is a very strict multiple testing correction method when using the moving average combined with the mean difference. This should be avoided because overly corrected results may introduce false negatives and disregard real hemimethylated sites, which can be rare and hard to identify.

**Supplemental Table 2**: Number of significant CpG sites selected based on different criteria.

| (a). CpG sites selected based on p-value only | | | | |
| --- | --- | --- | --- | --- |
|  | p<0.05 | p<0.05.d=25 | p<0.05.d=50 | p<0.05.d=100 |
| Normal | 102751 | 36521 | 26399 | 23641 |
| Tumor | 89326 | 33431 | 23471 | 20714 |
| (b) CpG sites selected based on both mean difference and p-value | | | | |
| MeanDiff  p-value | MeanDiff  p<0.05 | MeanDiff  p<0.05.d=25 | MeanDiff  p<0.05.d=50 | MeanDiff  p<0.05.d=100 |
| Normal | 7351 | 3354 | 2572 | 2329 |
| Tumor | 7330 | 3030 | 2303 | 2056 |

*The three rows in the top panel are the CpG sites selected based on p-value only; the last three rows in the bottom panel are the CpG sites selected based on both mean difference (MeanDiff) and p-value. The d=25, d=50, and d=100 column names correspond to the distance (in base pairs) that we used to calculate the moving-average of neighboring p-values. For example, “p<0.05.d=25” means the new p-value is obtained using the moving average of nearby CpG sites within d=25 base pairs on each side, and then we use p<0.05 to select significant CpG sites.*

The results of using comb-p FDR and SLK multiple testing corrections are shown in **Supplemental Table 3**. To simplify our analysis, we only show the results of analyzing chromosome 1 (chr1) CpG sites. **Supplemental Table 3** shows that the comb-p FDR correction is too strict because we have no significant hemimethylation sites identified at the 0.05 cutoff. The comb-p SLK correction can add more false negatives. For example, for the chr1 normal data, if we only use the raw p value<0.05, we identify 8,757 sites. If we use comb-p SLK, we identify 5,916 sites. Among these two lists of significant sites, there are only 2,602 sites identified by both selection methods. Therefore, using the comb-p SLK method may lead to the identification of 3,314 (5,916 – 2,602) false positive CpG sites for which the original data did not show a small p-value. This seems counterintuitive, but **Supplemental Table 4** explains why. In **Supplemental Table 4**, we run the comb-p based on the SLK correction (with default settings) for an example dataset of 100 sites provided in the comb-p web page. The table contains the top 10 rows and shows us that the SLK method identifies significant regions and then assigns all CpG sites in one region the same p-value. The SLK method may overcorrect the results and introduce both false negative and false positive sites. For example, **Supplemental Table 4** shows that the site with the start position 62,140 is not significant (with p-value = 0.79429). However, after the SLK correction, the modified p-value is 8.80E-05, which is a false-positive result.

**Supplemental table 3:**: The number of significant CpG sites selected for chromosome 1 (chr1).

| chr1 | p<0.05 | comb-p.FDR.p<0.05 | comb-p.SLK | SLK.Intersect |
| --- | --- | --- | --- | --- |
| Normal | 8757 | 0 | 5916 | 2602 |
| Tumor | 7209 | 0 | 4275 | 2155 |

*The “p<0.05” column describes the number of significant sites identified in chromosome 1. The “comb-p.FDR.p<0.05” column shows the number of significant sites after FDR correction. The “comb-p.SLK” column describes the number of significant sites after SLK correction. Lastly, the “SLK.Intersect” column shows the number of CpG sites that are significant both before and after the SLK correction.*

**Supplemental table 4:**: An example of the multiple testing correction p-value based on the SLK method.

| chr | start | end | p-value | SLK.p-value |
| --- | --- | --- | --- | --- |
| chr1 | 62082 | 62083 | 3.24E-08 | 8.80E-05 |
| chr1 | 62096 | 62097 | 3.94E-12 | 8.80E-05 |
| chr1 | 62112 | 62113 | 0.064892 | 8.80E-05 |
| chr1 | 62140 | 62141 | 0.79429 | 8.80E-05 |
| chr1 | 526852 | 526853 | 0.45423 | 0.50846 |
| chr1 | 526880 | 526881 | 0.13813 | 0.50846 |
| chr1 | 526889 | 526890 | 0.45877 | 0.50846 |
| chr1 | 526896 | 526897 | 0.73267 | 0.50846 |
| chr1 | 526910 | 526911 | 0.53226 | 0.50846 |
| chr1 | 526930 | 526931 | 0.58344 | 0.50846 |

*Each row corresponds to one CpG site. The first, second, and third columns describe the location of each CpG site. The “p-value” column gives the raw p-values with no correction. The last column, “SLK.p-value”, describes the p-values after SLK correction.*

After exploring various correction methods, we conclude that the mean difference plus p-value filtering method can produce meaningful and interpretable results when dealing with the multiple testing comparison problem for our hemimethylation analysis. As shown in **Supplemental Table 2**, applying the mean difference cutoff alone eliminates over 90% of p-values (e.g., 102,751 vs 7,351 for the normal, and 89,326 vs 7,330 for the tumor). In this context, a false positive means that the p-value suggests a statistical significance but there is not a practical (or biological) difference. False positive sites are unlikely to have large mean differences due to there being no significant biological difference. The false positive sites would be eliminated at a higher rate by the mean difference cutoff. Therefore, applying any additional corrections would be too strict and result in more false negatives. We have finally decided to use the p-value and mean difference to identify significant hemimethylation sites.

**References**

1. Pedersen BS, Schwartz DA, Yang IV, Kechris KJ: **Comb-p: software for combining, analyzing, grouping and correcting spatially correlated P-values**. *Bioinformatics* 2012, **28**(22):2986-2988.

2. Benjamini Y, Hochberg Y: **Controlling the false discovery rate: a practical and powerful approach to multiple testing**. *Journal of the Royal Statistical Society* 1995, **57**(1):289-300.

3. Kechris KJ, Biehs B, Kornberg TB: **Generalizing moving averages for tiling arrays using combined p-value statistics**. *Stat Appl Genet Mol Biol* 2010, **9**:Article29.
